# Hospital Expenditure and Its Reduction

**Published:** 1905-01-14

**Authors:** Henry Burdett, Sydney Holland


### HOSPITAL ADMINISTRATION.

### CONSTRUCTION AND ECONOMICS.

### HOSPITAL EXPENDITURE AND ITS REDUCTION.

By SIR HENRY BURDETT and the HON. SYDNEY HOLLAND.

An article headed "Hospital Waste: How to Save £3,000 a year," by Sir Henry Burdett, appeared in the *Daily Mail* of the 24th ultimo. Sir Henry commenced by a definition of the word "waste," to prevent the possibility of any one taking offence at its use in the sense in which he applied it to certain London hospitals. Sir Henry's main points were that the present system of free service by the medical profession requires considerable modification, and that the remedy is that every member of the medical profession attached to a hospital should receive payment for the medical service he renders. At a rough estimate he put down the cost to the London hospitals at £60,000 a year, or say about £6 10s. per occupied bed. The appointment of a medical superintendent in charge of every department, who could keep the expenditure practically uniform, should result in a saving of £20 per occupied bed. In this way the payment for all medical service would prove financially advantageous, and in fact, in Sir Henry's opinion, the London hospitals at the present time spend too little money in salaries. With the payment of the staff means must be found to prevent the present abuse of the voluntary hospitals by the admission of patients who can afford to pay for their treatment, and the enforcement of this and other economies would increase the efficiency of the hospitals in every department. In brief, Sir Henry maintained that economy can be readily enforced by modifying the system of contracts on the lines indicated by the Prince of Wales: by strengthening the administrative control by the appointment of a medical superintendent,* by the abolition of free medical attendance, the reform of the system of admission of patients so as to restrict their numbers and prevent abuse, and the modification of the system of accounts by accurately recording the actual cost of the out-patient department and the in-patient department, the whole expenditure upon each being kept distinct in the books for this purpose. These changes should reduce the cost per bed on an average at a London hospital to between £80 and £90 per year; if so, a saving in expenditure, after providing £60,000 for the payment of the medical staff, might be secured on the total expenditure of the London hospitals of something like £150,000 a year.

In the *Daily Mail* of the 27th ultimo Mr. Sydney Holland published a letter headed "A Reply to Sir Henry Burdett by the Hon. Sydney Holland, Chairman of the London and Poplar Hospitals." Mr. Holland objects to the payment of the staff, and declares that £6 10s. per occupied bed would work out at under £150 a year to each member of the staff, "a magnificent sum indeed with which to remunerate a man earning his thousands a year for visiting the hospital three times a week." He then states that at almost if not all hospitals with schools the staff do receive some pay either for attendance or teaching. He characterises Sir Henry's suggestion to appoint a medical superintendent as a fancy picture not to be taken seriously, and exclaims "Was ever such a proposition seriously put forward before?" "This paragon (*i.e.* the medical superintendent) is to be 'a competent, experienced and exceptionally able man,' and to obtain such a man, we hospital managers are to be restricted in our choice to a very limited class of men, men who, as a rule, have ever since they left school been tied down closely to the study of medicine, and who have necessarily been shut off in great measure from any other experience. What experience of organisation, or the management of men, of the maintaining of discipline, of provision buying, of dealing with estates, keeping accounts, of dealing with finance—in short, what experience at all, except in his own special line, does the education of a medical man ensure to him? And yet the organisation of a huge business like the London Hospital is to be handed over to a man only so educated." Mr. Holland then, under the heading "The Better Way," writes: "It seems to me that the best organisation of a hospital is to appoint at the head of each department a man skilled and experienced in the special business which that department has to carry on, and to put over these men a secretary or house governor with a good head for business, with organising power, and with a power of managing men, and over him to have a committee of management composed of men with every possible variety of experience." In conclusion, Mr. Holland regrets to find himself differing with Sir Henry Burdett so publicly, but the latter's article cannot be allowed to go unanswered, and "many people have asked me [Mr. Holland] to reply."

The *Daily Mail* of the 31st ult. contained the following reply from Sir Henry Burdett:—

The Chairman of the London Hospital does not touch my argument at all. An obvious clerical error whereby the word "unduly" was substituted for "duly" is made the most of, and then Mr. Holland questions the proposals (1) to pay the medical staff, and (2) to appoint a medical superintendent. The payment of the staff would be an act of justice to the whole medical profession, would promote economy, and help the hospitals. I estimate that the majority of the staff at a great hospital would receive £300 a year each, a sum not "absurdly inadequate." Substantial remuneration should be provided for about £60,000 a year. How many doctors are earning "thousands a year" in London?

As to (2), a large number of medical men are medical superintendents to-day in entire charge of great hospitals

* See The Hospital for Nov. 26, Dec. 3, Dec. 10, 1904.

and asylums who, judged by results, prove Mr. Holland's statement to be an ill-considered libel. Some of the ablest doctors have been or are medical superintendents—to wit, Sir James Crichton-Browne and Sir Cooper Perry. The work has great attraction for able administrators, and such men would save their salary, whatever the amount, many times over. Adequate salaries are essential to economical administration. My views on this point are enforced by upwards of twelve years' experience as a house governor or lay superintendent of more than one hospital. An experience mainly confined to the London Hospital, where the medical staff have no voice in the management, must be necessarily incomplete and unreliable on the points at issue.

My position is that the present contributions to London hospitals can be made adequate to pay for all the work they can properly undertake. To this end adequate control of the expenditure is essential however. I am convinced, for instance, that a medical superintendent could reduce the expenditure of the London hospitals by so large a sum as to astonish Mr. Sydney Holland and all who believe that the present control is thorough and sufficient.

In the *Daily Mail* of the 4th inst. Mr. Sydney Holland writes as follows :—

Sir Henry Burdett repeats his estimate that £6 10s. per occupied bed would be sufficient to pay the medical staff of a hospital an adequate salary—a salary "substantial" enough, as he calls it, to reduce them from the position of honorary officers into that of paid officials. I replied that this would work out at the magnificent sum of £150 a year, and he replies that it would work out at £300 a year, which he considers substantial.

Take the London. There were 676 occupied beds in 1903. The sum to be divided among the 34 members of the staff would be, therefore, £4,384 = £129 each.

So, to carry out Sir Henry's suggestion—namely, to give the staff this substantial salary of £300 a year—we shall need at once to increase our expenditure not by £4,384, but by £10,200! And this is the serious advice of a man who writes an article advising all hospitals how to manage their businesses.

It will be seen that this last letter of Mr. Holland contains at least two misstatements : (1) Sir Henry Burdett did not repeat in his letter of 31st ultimo his estimate of £6 10s. per bed ; (2) Sir Henry's estimate was that *the majority of the staff at a great hospital would receive* £300 *a year each*, whilst Mr. Holland attributes to him the statement that his estimate *would give the staff* (*i.e.* the whole staff) *this substantial salary of* £300 *a year*. As to (1) it will be noticed that Sir Henry Burdett explained that his was a rough estimate, and the cost per occupied bed was an obvious calculation based on the total number of beds divided into the estimated cost (£60,000) for medical service. Sir Henry's calculation, it will be seen, bore no relation to the apportionment of the total sum amongst the hospitals, or the members of each staff. On the latter point his statement is that his estimate should give the majority of the staff at a great hospital £300 a year each. As to Mr. Holland's own calculations in regard to the London Hospital, of which he is chairman, we should rejoice to learn that the medical staff of the London were to be paid from £8,000 to £10,000 a year, and to have justice done them by being admitted to the management of its affairs, for then we believe that a brief experience would show that a material saving in expenditure would result.

